# Caloric restriction impacts plasma microRNAs in rhesus monkeys

**DOI:** 10.1111/acel.12636

**Published:** 2017-07-05

**Authors:** Augusto Schneider, Joseph M. Dhahbi, Hani Atamna, Josef P. Clark, Ricki J. Colman, Rozalyn M. Anderson

**Affiliations:** ^1^ Faculdade de Nutrição Universidade Federal de Pelotas Pelotas‐RS 96010‐610 Brazil; ^2^ College of Medicine Burnett School of Biomedical Sciences University of Central Florida Orlando FL 32827 USA; ^3^ College of Medicine California University of Science and Medicine Colton CA 92324 USA; ^4^ Department of Medicine University of Wisconsin Madison WI 53705 USA; ^5^ Wisconsin National Primate Research Center Madison WI 53706 USA; ^6^ GRECC William S. Middleton Memorial Veterans Hospital Madison WI 53705 USA

**Keywords:** aging, caloric restriction, microRNA, miR‐125a‐5p, rhesus monkeys

## Abstract

Caloric restriction (CR) is one of the most robust interventions shown to delay aging in diverse species, including rhesus monkeys (*Macaca mulatta*). Identification of factors involved in CR brings a promise of translatability to human health and aging. Here, we show that CR induced a profound change in abundance of circulating microRNAs (miRNAs) linked to growth and insulin signaling pathway, suggesting that miRNAs are involved in CR's mechanisms of action in primates. Deep sequencing of plasma RNA extracts enriched for short species revealed a total of 243 unique species of miRNAs including 47 novel species. Approximately 70% of the plasma miRNAs detected were conserved between rhesus monkeys and humans. CR induced or repressed 24 known and 10 novel miRNA species. Regression analysis revealed correlations between bodyweight, adiposity, and insulin sensitivity for 10 of the CR‐regulated known miRNAs. Sequence alignment and target identification for these 10 miRNAs identify a role in signaling downstream of the insulin receptor. The highly abundant miR‐125a‐5p correlated positively with adiposity and negatively with insulin sensitivity and was negatively regulated by CR. Putative target pathways of CR‐associated miRNAs were highly enriched for growth and insulin signaling that have previously been implicated in delayed aging. Clustering analysis further pointed to CR‐induced miRNA regulation of ribosomal, mitochondrial, and spliceosomal pathways. These data are consistent with a model where CR recruits miRNA‐based homeostatic mechanisms to coordinate a program of delayed aging.

Rhesus monkeys share marked similarities with humans including many age‐related phenotypes and clinical conditions. Adult onset CR delays morbidity and mortality in rhesus monkeys, with a 2.9‐fold increased risk of disease (Colman *et al*., 2009) and a 2.9‐fold increased risk death from age‐related causes in controls compared to CR (Colman *et al*., 2014). A newly identified potential mechanism of CR involves a change in tissue abundance of microRNAs (miRNAs) (Mori *et al*., 2012; Mercken *et al*., 2013). miRNAs are short small noncoding RNAs of about 22 nucleotides in length that regulate mRNA translation and stability (Bartel, 2004). Age‐related changes in circulating miRNAs have been described in rodents and in humans (Dhahbi, 2014). Increasing evidence supports a role for miRNAs in intercellular communication (Cortez *et al*., 2011; Chen *et al*., 2012), and a recent study reported that adipose‐derived circulating miRNAs influence gene expression in distal tissues (Thomou *et al*., 2017). Here, we investigated the impact of long‐term CR on plasma miRNAs in rhesus monkeys.

## Results and discussion

This study was conducted using fasted plasma from 10 male rhesus monkeys of ~25 years of age on control or CR diets for 17 years. Bodyweight and adiposity were lower in CR monkeys compared to controls, and insulin sensitivity was increased (*P* < 0.05; Table S1, Supporting information). Bodyweight and percent body fat were positively correlated with each other (*r* = 0.92, *P* = 0.0001), and both tended to be negatively correlated to insulin sensitivity (*P* = 0.06; *P* = 0.07, respectively). Total RNA was extracted from plasma, enriched for RNAs of <200 nucleotides, and sequenced at 1x50 base pair reads. On average, 11 676,740 ± 658 741 reads per sample were obtained. miRNAs represented ~15% of the aligned reads. Known and novel miRNAs were identified using miRDeep2. Genomic coordinates were assigned, and novel miRNAs were assigned by direct alignment to MacaM/rhemac8 (Zimin *et al*., 2014).

**Figure 1:**
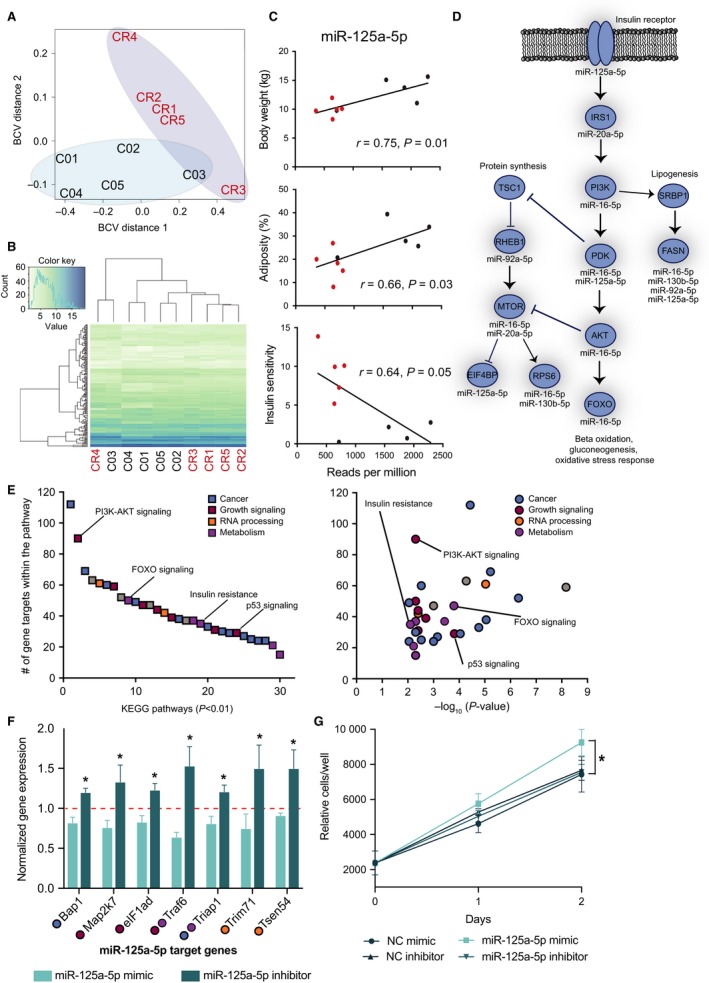
Impact of CR on circulating miRNAs. (A) Principal component analysis of bootstrap smoothed cross‐validation estimates, and (B) unsupervised hierarchical clustering of total detected plasma miRNAs. (C) Regression analysis for miRNA125a‐5p against biometric and insulin sensitivity data, (D) CR‐regulated miRNAs correlating with physiological outcomes of CR, (E) putative target pathways responsive to CR‐induced changes in miRNAs. (F) Expression of indicated genes contained within pathways from E in mouse NIH‐3T3‐L1 pre‐adipocytes transfected with miR‐125a‐5p mimic, inhibitor, or corresponding negative control miRNAs (*P < 0.05 between normalized mimic‐ and inhibitor‐treated cells, *n* = 3 experiments). (G) Growth curves for cells transfected with miR‐125a‐5p mimic, inhibitor, or negative control miRNA (*n* = 5). Data are shown as average ± SD (*P < 0.05).

In total, 243 unique plasma miRNA sequences were detected, of which 196 had been previously described (TablesS2 and S3, Supporting information). Twenty‐four distinct known miRNAs from 18 different miRNA gene families were significantly differentially expressed between control and CR monkeys (Table 1). Among these are miRNAs linked to obesity including miR‐21, miR‐143, miR‐125a, and miR‐125b (Hilton *et al*., 2013); and miR‐21, miR‐20a, miR‐125b, miR‐16, miR‐224 previously linked to cancer (Calin & Croce, 2006). Applying principal component analysis, sample dispersion was clustered by group and separated by diet (Fig. 1A). Unsupervised hierarchical clustering of all detected miRNA species for each plasma sample indicated a remarkably low level of sample variability (Fig. 1B). Nine novel species of miRNA were significantly differentially expressed between control and CR monkeys (Table S3, Supporting information). Approximately 70% of the plasma miRNAs were conserved between rhesus and human (Williams *et al*., 2013). Of the novel miRNAs, 31 (66%) were previously identified in human mirbase v.20 (GRCh37.p5) by seed sequence. Regression analysis showed that 10 of the differentially expressed known miRNAs significantly correlated with bodyweight, body fat content, or insulin sensitivity (Table S4, Supporting information) and one, miR‐125a‐5p, was significantly correlated to all three parameters (Fig. 1C). The main target genes from this group of 10 miRNAs fall within the insulin signaling pathway (Fig. 1D). A further 30 known miRNAs that were not differentially expressed between groups were correlated with at least one of the above three parameters (Table S5, Supporting information). As decreases in bodyweight and adiposity are an expected outcome of CR, we performed a one‐way analysis of covariance and found that miR‐125a‐5p, miR‐130a‐5p and miR‐143‐5p were affected by the diet (*P* < 0.05) even after adjusting for bodyweight or adiposity. The expression of miR‐130b‐5p, miR‐16‐5p, and miR‐20a‐5p tended to be affected by the diet (*P*‐value between 0.05 and 0.07), while changes in expression of miR‐337‐5p, miR‐411‐5p, miR‐6529‐5p, and miR‐92a‐5p were dependent on bodyweight or adiposity. These data indicate that while levels of some circulating species of miRNA are directly related to adiposity, others appear to be influenced by additional mechanisms.

**Table 1:** Circulating microRNAs (miRNAs) differentially expressed between calorie‐restricted and control rhesus monkeys

*Macaca mulatta* miRNA^a^	miRNA gene family	miRDeep2 score^b^	Estimated probability^c^	Control^d^	Calorie restriction^e^	FC^f^	*P* value^f^
Downregulated							
mml‐miR‐486‐5p	miR‐486	2 700 000	0.94 ± 0.03	362 234.9 ± 75903.4	159 672.7 ± 24491.3	−2.3	0.0094
mml‐miR‐92a‐3p	miR‐25	650 000	0.94 ± 0.03	67 079.2 ± 8223.8	40 718.2 ± 2180.5	−1.6	0.0161
mml‐miR‐16‐5p	miR‐15	180 000	0.94 ± 0.03	17 829.1 ± 2373.4	10 801.1 ± 685.6	−1.7	0.0199
mml‐miR‐125a‐5p	miR‐10	5.5	0.93 ± 0.02	1712.5 ± 276.4	632.7 ± 75.5	−2.7	0.0003
mml‐miR‐125b‐5p	miR‐10	5.3	0.93 ± 0.02	145.9 ± 25.4	74.1 ± 15.4	−2.0	0.0306
mml‐miR‐143‐5p	miR‐143	5.5	0.93 ± 0.02	109.6 ± 37.1	31.7 ± 6.4	−3.5	0.0046
mml‐miR‐106b‐5p	miR‐17	2500	0.94 ± 0.03	77.5 ± 14.8	40.4 ± 3.7	−1.9	0.0133
mml‐miR‐20a‐5p	miR‐17	680	0.94 ± 0.03	66.0 ± 4.2	40.5 ± 4.1	−1.6	0.0227
mml‐miR‐133c‐5p	miR‐133	320	0.94 ± 0.03	53.2 ± 13.2	17.6 ± 5.7	−3.0	0.0057
mml‐miR‐133b‐5p	miR‐133	5	0.93 ± 0.02	35.8 ± 9	11.9 ± 3.9	−3.0	0.0065
mml‐miR‐182	miR‐182	5.4	0.93 ± 0.02	7.7 ± 1.6	3.0 ± 1	−2.6	0.0283
mml‐miR‐224‐5p	miR‐224	5.4	0.93 ± 0.02	5.6 ± 2.7	1.3 ± 0.4	−4.3	0.0228
Upregulated							
mml‐miR‐6529‐5p	miR‐6529	71 000	0.94 ± 0.03	2533.6 ± 793.4	5095.8 ± 694.2	2.0	0.0295
mml‐miR‐21‐5p	miR‐21	46 000	0.94 ± 0.03	1987.4 ± 253.9	3108.8 ± 312.9	1.6	0.0420
mml‐miR‐340‐5p	miR‐340	3700	0.94 ± 0.03	145.8 ± 25.1	251.5 ± 41	1.7	0.0456
mml‐miR‐130a‐5p	miR‐130	5.4	0.93 ± 0.02	99.0 ± 12.5	191.5 ± 13.7	1.9	0.0023
mml‐miR‐1260b	miR‐1260b	5.6	0.93 ± 0.02	62.4 ± 22.4	142.6 ± 30.1	2.3	0.0337
mml‐miR‐130b‐5p	miR‐130	2300	0.94 ± 0.03	40.5 ± 6.2	75.3 ± 9.4	1.8	0.0160
mml‐miR‐411‐5p	miR‐379	1900	0.94 ± 0.03	41.0 ± 4.6	72.0 ± 12.4	1.8	0.0287
mml‐miR‐598‐5p	miR‐598	5.2	0.93 ± 0.02	7.7 ± 2	22.1 ± 8.2	2.8	0.0316
mml‐miR‐500a‐5p	miR‐500	170	0.94 ± 0.03	3.4 ± 0.8	17.8 ± 3.9	5.2	0.0001
mml‐miR‐501‐5p	miR‐500	170	0.94 ± 0.03	3.4 ± 0.8	17.8 ± 3.9	5.2	0.0001
mml‐miR‐122a‐5p	miR‐122	150	0.94 ± 0.03	5.5 ± 0.7	15.6 ± 5.5	2.7	0.0132
mml‐miR‐337‐5p	miR‐337	230	0.94 ± 0.03	3.4 ± 0.9	7.6 ± 1.3	2.1	0.0372

a Names of mature miRNAs of *Macaca mulatta* in miRbase v.20 (MMUL1.0) that match precursor sequences predicted by miRDeep2 and have an randfold *P*‐value < 0.05.

b The miRDeep2 score represents the log‐odds probability of a sequence being genuine miRNA precursor vs. the probability that it is a background hairpin.

c The estimated probability that the miRNA candidate is a true positive.

d Average miRNA read counts‐per‐million computed for each group and taking into account the estimated dispersions and the libraries sizes.

e It represents a measure of the expression level of the miRNA in the indicated sample.

f Fold change and *P* value for differential abundance were computed by EdgeR from pairwise comparisons for each miRNA between the control and CR groups.

A search of miRecords, miRTarBase, and Tarbase identified 2,935 target genes of the known miRNAs that were differentially expressed with CR. Pathway analysis was conducted using both KEGG and GO platforms, cut‐off criteria of >10 target genes, and calculated Benjamini–Hochberg‐corrected *P *< 0.01 applied (TablesS6 and S7, Supporting information). The KEGG‐enriched pathways included several pathways known for their role on aging, such as PI3k‐Akt (Fig. S1, Supporting information), FoxO, p53, insulin signaling pathways, and multiple cancer pathways (Fig. 1E). The enriched GO terms included translation, cell cycle, spliceosome, DNA damage, and cancer. Using clustering analysis of the outcomes of all pathway analyses, clusters related to cell cycle and cellular signaling by phosphorylation were highly enriched in the differentially expressed miRNA gene target pool (Table S8, Supporting information). Other pathways included ribosome‐, mitochondria‐, and spliceosome‐related pathways. Interestingly, the former two pathways were identified as major components of a conserved tissue type‐independent transcriptional signature of CR (Barger *et al*., 2015). We next used a cell culture model to functionally validate target genes contained within the enriched pathways (Fig. 1E), focusing on genes that are predicted to be conserved from mouse to humans. 3T3‐L1 pre‐adipocytes were treated with synthetic miRNAs designed to mimic or inhibit miR‐125a‐5p (Fig. 1F). Moreover, pre‐adipocytes transfected with the miR‐125a‐5p mimic acquired a significant growth advantage over inhibitor and negative control‐transfected cells (Fig. 1G), confirming a role for miR‐125a‐5p in growth promotion *in vivo*.

Our data are consistent with the concept that miRNAs are evolutionary conserved in specific lineages such as the primates (Kamanu *et al*., 2013). Plasma miRNA expression levels are similar for humans and rhesus (Noren Hooten *et al*., 2013), and homology between species is 90–100% (Yue *et al*., 2008). Long‐term CR changed the abundance of plasma miRNAs that are predicted to target multiple pathways, including insulin and growth signaling pathways linked to cancer and to aging (Bartke, 2011), and miR‐125a‐5p, previously linked to obesity and insulin resistance (Herrera *et al*., 2009; Diawara *et al*., 2014), was decreased significantly by CR. Taken together, our study suggests that RNA‐based homeostatic mechanisms are recruited by CR to coordinate delayed aging.

## Funding

This work was supported by NIH/NIA AG040178, AG047358, and the Glenn Foundation for Medical Research. JPC is a T32 Fellow NIH/NIA AG000213. The study was conducted with the use of resources and facilities at the William S. Middleton Memorial Veterans Hospital, Madison, WI.

## Conflict of interest

The authors report no conflict of interest financial or otherwise.

## Supporting information


**Fig. S1** PI3K‐AKT signaling pathway.
**Table S1** Age, total, lean and fat body weight of calorie restricted and control rhesus monkeys.
**Table S2** Abundance (in reads per million) of all circulating microRNAs (miRNAs) detected in calorie restricted and control rhesus.
**Table S3** Abundance (in reads per million) of all circulating novel microRNAs (miRNAs) between CR and control rhesus monkeys.
**Table S4** microRNAs differentially expressed between control and CR rhesus monkeys and with significant correlation with body weight, fat percentage or insulin sensitivity.
**Table S5** Known and novel microRNAs non‐responsive to CR, but with significant correlation with body weight, fat percentage or insulin.
**Table S6** Enriched KEGG pathways for the genes targeted by miRNA differentially expressed between CR and control rhesus monkeys.
**Table S7** Enriched gene ontology (GO) terms for molecular function and biological process for the genes targeted by miRNA differentially expressed between calorie restricted and control rhesus monkeys.
**Table S8** Functional Annotation Cluster reporting terms for which contributing terms were independently significant for the genes targeted by miRNA differentially expressed between CR and control rhesus monkeys.
**Appendix S1** Methods.
