# Supplementary material for: Caloric restriction impacts plasma microRNAs in rhesus monkeys
Source: Aging Cell. 2017 Jul 5;16(5):1200–3. doi: 10.1111/acel.12636 (PMC5595684; doi:10.1111/acel.12636)
Supplement: Supplementary file 1 — Fig. S1 PI3K‐AKT signaling pathway. Table S1 Age, total, lean and fat body weight of calorie restricted and control rhesus monkeys. Table S2 Abundance (in reads per million) of all circulating microRNAs (miRNAs) detected in calorie restricted and control rhesus. Table S3 Abundance (in reads per million) of all circulating novel microRNAs (miRNAs) between CR and control rhesus monkeys. Table S4 microRNAs differentially expressed between control and CR rhesus monkeys and with significant correlation with body weight, fat percentage or insulin sensitivity. Table S5 Known and novel microRNAs non‐responsive to CR, but with significant correlation with body weight, fat percentage or insulin. Table S6 Enriched KEGG pathways for the genes targeted by miRNA differentially expressed between CR and control rhesus monkeys. Table S7 Enriched gene ontology (GO) terms for molecular function and biological process for the genes targeted by miRNA differentially expressed between calorie restricted and control rhesus monkeys. Table S8 Functional Annotation Cluster reporting terms for which contributing terms were independently significant for the genes targeted by miRNA differentially expressed between CR and control rhesus monkeys. Appendix S1 Methods. [file ACEL-16-1200-s001.pdf]

Supplementary Material

- Methods
- Tables S1-8
- Figure S1

Methods

Animals and diets Animals were maintained in accordance to guidelines for the ethical care and treatment of animals as approved by the Institutional Animal Care and Use Committee of the Graduate School of the University of Wisconsin-Madison. This study involved 10 male monkeys from the Aging and Caloric Restriction study (Ramsey *et al.* 2000). All animals were of Indian origin and were born at the WNPRC. Prior to the start of the study no animals had any clinical or experimental history that would be expected to affect disease susceptibility or mortality. The animals were randomized to control or CR diets taking into consideration baseline food intake, body weight and age. CR was implemented at 10% reduction per month over a 3-month period to reach the desired 30% restriction. CR intake was individually determined from baseline measures. Animals were fed a semi-purified, nutritionally fortified, low fat diet containing 15% protein and 10% fat. Complete clinical and experimental histories are maintained for each animal. In the current study, 5 control and 5 CR monkeys were 25 ± 0.4 years of age at the time of specimen collection and had been enrolled in the study for 17 years.

Plasma RNA isolation and small RNA library construction Plasma samples drawn >3hrs following glucose infusion during a frequently sampled intravenous glucose tolerance test, a time-point when baseline measures of insulin and glucose were reestablished (Ramsey *et al.* 2000; Gresl *et al.* 2001). Isolation of total RNA, including small RNA, was performed with the miRNeasy kit (#217004, Qiagen, Hilden, Germany) according to the manufacturer's protocol with the following alterations: 1 mL of Qiazol reagent was mixed with 0.2 mL of plasma, the entire aqueous phase was loaded onto a single column from the MinElute Cleanup Kit (#74204, Qiagen, Hilden, Germany), and RNA was eluted in 20 µL of RNase-free water. One fourth (5 µL) of the RNA isolated from each plasma sample was used to construct sequencing libraries with the Illumina TruSeq Small RNA Sample Prep Kit (#RS-200-0012, Illumina, San Diego, CA), following the manufacturer's protocol. Briefly, 3′ and 5′ adapters were sequentially ligated to small RNA molecules and the obtained ligation products were subjected to a reverse transcription reaction to create single stranded cDNA. To selectively enrich fragments with adapter molecules on both ends, the cDNA was amplified with 15 PCR cycles using a common primer and a primer containing an index tag to allow sample multiplexing. The amplified cDNA constructs were gel purified, and validated by checking the size, purity, and concentration of the amplicons on the Agilent Bioanalyzer High Sensitivity DNA chip (#5067-4626, Genomics Agilent, Santa Clara, CA). The libraries were pooled in equimolar amounts, and sequenced on an Illumina HiSeq 2000 instrument to generate 50-base reads.

MiRDeep2 analysis of sequencing reads Sequencing reads were analyzed with miRDeep2 (Friedlander *et al.* 2012), a probabilistic algorithm based on the miRNA biogenesis model and designed to detect miRNAs from deep sequencing reads. Briefly, the miRDeep2 algorithm aligns reads to potential hairpin structures in a manner consistent with Dicer processing and assigns scores that measure the probability that hairpins are true miRNA precursors. For the purpose of analyzing the sequenced miRNAs, the known miRNA input was from miRBase v.20 (Kozomara & Griffiths-Jones 2014) with *Homo sapiens* designated as the related species. Detected miRNAs were annotated as rheMac8 assembly genomic coordinates (Nov. 2015; GenBank Accession ID: GCF_000772875.2). Seed sequences of novel miRNAs were queried by alignment to mature miRNAs of *Homo sapiens* documented in miRbase v.20 (GRCh37.p5).

Statistical analysis of differential circulating small RNA levels In addition to identifying mature known and novel miRNAs in deep sequenced small RNA samples, miRDeep2 also generates expression values for the detected miRNAs. The expression miRNA data produced by miRDeep2 were analyzed with the Bioconductor package edgeR (Robinson *et al.* 2010) to quantify the changes in the levels of circulating miRNAs between control and CR groups. The algorithm of edgeR fits a negative binomial model to the count data, estimates dispersion, and measures differences using the generalized linear model likelihood ratio test. Only miRNAs present in at least 50% of the samples with more than 3 reads per million were further considered for analysis. Unsupervised hierarchical clustering for the 196 known detected miRNAs was performed using the HeatMap2 function in R (3.2.2) and the principal component analysis was performed using the plotMDS function from edgeR also in R. Correlations between individual miRNAs and biometric and insulin sensitivity measures were calculated using linear regression, r values and unadjusted p values are reported. Additionally, One-way ANOVA analysis of covariance was performed to test the effect of bodyweight and adiposity on miRNA expression. Here we report an adjusted p-value considering bodyweight and adiposity as covariates for all miRNAs that had a significant correlation with these parameters.

Prediction of miRNA target genes and their functional analysis The human miRNA orthologs were used for predicting gene targets using the R (3.2.2) script multiMiR (Ru *et al.* 2014). The databases miRecords (Xiao *et al.* 2009), miRTarBase (Chou *et al.* 2016) and Tarbase (Vlachos *et al.* 2015) were used to predict the genes that are experimentally validated targets of the differentially expressed miRNAs. The predicted target genes were analyzed with the Functional Annotation Chart tool from DAVID (Huang da *et al.* 2009) to determine which KEGG pathways (Kanehisa & Goto 2000; Kanehisa *et al.* 2016) and gene ontology (GO) biological processes are specifically enriched in the gene list. This tool measures the similarities among KEGG pathways and GO terms based on the extent of the associated genes. Pathways and terms with calculated Benjamini-Hochberg corrected p-Value lower than 0.01 and containing more than 10 target genes were considered as significant. In addition, the Functional Annotation Clustering tool from DAVID was used to identify functionally related

groups implicated in the CR response. The Enrichment Score represents the geometric mean (in log scale) of the group members p-values for each annotation cluster.

Cell culture, transfections, RT-qPCR, and CyQuant assay Mouse NIH-3T3-L1 pre-adipocytes were cultured in DMEM supplemented with 10% bovine serum and 1% Pen/Strep, and cultured at 37ºC, 5% CO_2. Prior to all assays, cells were passaged in log phase for one doubling. To validate miR-125a-5p gene targeting, cells were plated at $10.5x10^4$ cells/cm^2 in one well of a 6-well plate. 24 hrs later, cells were transfected with either a *mir*Vana (Ambion) miR-125a-5p mimic (cat #MC12561), inhibitor (cat #MH12561), or appropriate negative control (cat #4464058 or cat #4464077) at 30 nM using Lipofectamine 2000. 24 hrs later total RNA was harvested in TRIzol (Invitrogen), isolated and cDNA was made using High-Capacity cDNA reverse transcriptase kit (Applied Biosystems). Real-time qPCR detection was performed using iTaq sybr green (Applied Biosystems). The following primer pairs were used to detect the subsequent genes (all primers are listed 5'→3'):
Bap1: gggtgcaagtggaggagatc, tcacagacgtgtcatccacc;
eIF1ad: gtgaaggctgagatctcttttgtg, acagctgtggctcagcag;
Map2k7: gaataagcgcattttgatggacc, ccctgcattcgtttcttcagc;
Traf6: tctgcttgatggctttacgg, cagtatttcattgtcaactgggc;
Triap1: gctggtttgctgagaagttcc, gcaggcaggtcaagaggag;
Trim71: cattctctcggtgttcccg, cagctggatggtgagtgc;
Tsen54: ggcataccagctgctgct, ccatccaaattaagctgccgc;
To measure the impact of miR-125a-5p on growth, cells were plated at $5.3x10^3$ cells/cm^2 in a 96 well plate, and 24 hrs later were transfected with mimic, inhibitor or appropriate negative control miRNA at 30 nM using Lipofectamine 2000. At both 24 and 48 hrs later, cells were harvested, and quantitation was performed using CyQuant proliferation assay (Invitrogen) according to manufacture's protocol.

Methods references

Chou CH, et al. (2016). miRTarBase 2016: updates to the experimentally validated miRNA-target interactions database. *Nucleic acids research*. **44**, D239-247.

Friedlander MR, et al. (2012). miRDeep2 accurately identifies known and hundreds of novel microRNA genes in seven animal clades. *Nucleic acids research*. **40**, 37-52.

Gresl TA, et al. (2001). Dietary restriction and glucose regulation in aging rhesus monkeys: a follow-up report at 8.5 yr. *American journal of physiology. Endocrinology and metabolism*. **281**, E757-765.

Huang da W, et al. (2009). Systematic and integrative analysis of large gene lists using DAVID bioinformatics resources. *Nature protocols*. **4**, 44-57.

Kanehisa M , Goto S (2000). KEGG: kyoto encyclopedia of genes and genomes. *Nuclei Acid Research*. **28**, 27-30.

Kanehisa M, et al. (2016). KEGG as a reference resource for gene and protein annotation. *Nuclei Acid Research*. **44**, D457-462.

Kozomara A , Griffiths-Jones S (2014). miRBase: annotating high confidence microRNAs using deep sequencing data. *Nucleic acids research*. **42**, D68-73.

Ramsey JJ, et al. (2000). Dietary restriction and aging in rhesus monkeys: the University of Wisconsin study. *Exp Gerontol*. **35**, 1131-1149.

Robinson MD, et al. (2010). edgeR: a Bioconductor package for differential expression analysis of digital gene expression data. *Bioinformatics*. **26**, 139-140.

Ru Y, et al. (2014). The multiMiR R package and database: integration of microRNA-target interactions along with their disease and drug associations. *Nucleic acids research*. **42**, e133.

Vlachos IS, et al. (2015). DIANA-miRPath v3.0: deciphering microRNA function with experimental support. *Nucleic acids research*. **43**, W460-466.

Xiao F, et al. (2009). miRecords: an integrated resource for microRNA-target interactions. *Nucleic acids research*. **37**, D105-110.

Table S1 – Age, total, lean and fat body weight of calorie restricted and control rhesus monkeys.

Parameter	Control	CR	p value*
Age, y	25.6 ± 0.2	25.5 ± 0.2	0.59
Body weight, kg	13.1 ± 1.1	10.0 ± 0.6	0.04
Fat (%)	29.5 ± 3.3	17.7 ± 3.1	0.03
Lean (%)	69.9 ± 3.5	83.5 ± 3.3	0.02
Insulin sensitivity	1.5 ± 0.5	9.3 ± 1.5	0.003
Interleukin 6, ng/mL	3.7 ± 1.1	2.0 ± 0.4	0.19

* p values calculated using Students t test

Table S2 – Abundance (in reads per million) of all circulating microRNAs (miRNAs) detected in calorie restricted and control rhesus.

miRBase miRNA[1]	miRDeep2 score[2]	Estimated probability[3]	Control[4]	CR[4]
mml-let-7a-5p	6.58E+03	94 ± 3%	577.8 ± 57.4	601.7 ± 17.4
mml-let-7b-5p	2.85E+03	94 ± 3%	152.9 ± 18.3	155.1 ± 33.8
mml-let-7c-5p	2.50E+02	94 ± 3%	21.6 ± 3.3	16.4 ± 2
mml-let-7e-5p	2.76E+02	94 ± 3%	23.4 ± 3.1	23 ± 4.4
mml-let-7f-5p	1.54E+04	94 ± 3%	1131.3 ± 110.1	1307.7 ± 60.8
mml-let-7g-5p	2.60E+03	94 ± 3%	214.7 ± 10.9	162.2 ± 12.4
mml-let-7i-5p	4.59E+03	94 ± 3%	286.5 ± 37.8	288.8 ± 31.1
mml-miR-100-5p	4.9	85 ± 3%	204.1 ± 39.4	160.3 ± 67.1
mml-miR-101-1-5p	1.8	57 ± 3%	166.8 ± 18.4	167.8 ± 5.2
mml-miR-101-2-5p	0.9	31 ± 4%	166.8 ± 18.4	167.8 ± 5.2
mml-miR-103-3p	4.7	85 ± 3%	329.1 ± 62.6	400.3 ± 88.3
mml-miR-103-5p	4.7	85 ± 3%	329.1 ± 62.6	400.3 ± 88.3
mml-miR-106b-5p	2.58E+03	94 ± 3%	77.5 ± 14.8	40.4 ± 3.7
mml-miR-107-5p	4.7	85 ± 3%	329.1 ± 62.6	400.3 ± 88.3
mml-miR-10a-5p	6.56E+04	94 ± 3%	5552.9 ± 484.1	4799.3 ± 462.6
mml-miR-10b-5p	1.11E+05	94 ± 3%	15481 ± 2967.5	8219.1 ± 2105.5
mml-miR-1180-5p	4.12E+02	94 ± 3%	29.8 ± 9.2	19.4 ± 4.2
mml-miR-1185-5p	2.51E+02	94 ± 3%	3.6 ± 1	6.4 ± 2.7
mml-miR-122a-5p	1.56E+02	94 ± 3%	5.5 ± 0.7	15.6 ± 5.5
mml-miR-1249	5.1	93 ± 2%	2.1 ± 1.1	6.7 ± 2.5
mml-miR-125a-5p	5.5	93 ± 2%	1712.5 ± 276.4	632.7 ± 75.5
mml-miR-125b-5p	5.3	93 ± 2%	145.9 ± 25.4	74.1 ± 15.4
mml-miR-1260b	5.6	93 ± 2%	62.4 ± 22.4	142.6 ± 30.1
mml-miR-127-5p	5.1	93 ± 2%	389.3 ± 122.2	538.4 ± 158.8
mml-miR-1271-5p	6.84E+02	94 ± 3%	41.9 ± 12.5	44.2 ± 3.7
mml-miR-1277	5.53E+01	94 ± 3%	2.1 ± 0.9	3.6 ± 0.9
mml-miR-1284	0.9	31 ± 4%	3.9 ± 0.9	3 ± 0.7
mml-miR-128a-5p	9.13E+03	94 ± 3%	566.6 ± 73.3	520.3 ± 18.2

mml-miR-128b-5p	1.4	57 ± 3%	566.6 ± 73.3	520.3 ± 18.2
mml-miR-1296-5p	5.5	93 ± 2%	9.8 ± 2.3	8.2 ± 1.6
mml-miR-1306-5p	1.97E+02	94 ± 3%	10.6 ± 2.7	14.5 ± 1.7
mml-miR-130a-5p	5.4	93 ± 2%	99 ± 12.5	191.5 ± 13.7
mml-miR-130b-5p	2.36E+03	94 ± 3%	40.5 ± 6.2	75.3 ± 9.4
mml-miR-133b-5p	5	93 ± 2%	35.8 ± 9	11.9 ± 3.9
mml-miR-133c-5p	3.20E+02	94 ± 3%	53.2 ± 13.2	17.6 ± 5.7
mml-miR-134-5p	1.14E+03	94 ± 3%	48.2 ± 15	74.1 ± 10.7
mml-miR-136	6.25E+03	94 ± 3%	199.2 ± 57.2	400.3 ± 78.2
mml-miR-139-5p	5.83E+01	94 ± 3%	8.2 ± 2.6	3.7 ± 1
mml-miR-140-5p	2.1	53 ± 5%	1585.6 ± 167.4	1999.5 ± 194.1
mml-miR-141-5p	5.6	93 ± 2%	29.2 ± 6.7	17.3 ± 3.7
mml-miR-142-5p	9.63E+04	94 ± 3%	6516.5 ± 468.5	6688.7 ± 569.4
mml-miR-143-5p	5.5	93 ± 2%	109.6 ± 37.1	31.7 ± 6.4
mml-miR-144	1.49E+03	94 ± 3%	78.4 ± 17	51.7 ± 8.9
mml-miR-145-5p	9.66E+01	94 ± 3%	8.9 ± 2.3	5.1 ± 1.3
mml-miR-146a-5p	1.43E+03	94 ± 3%	101.9 ± 13.3	92.6 ± 6.1
mml-miR-146b-5p	1.54E+02	94 ± 3%	11.3 ± 1.5	8 ± 1.1
mml-miR-148a-3p	1	57 ± 3%	10044.7 ± 3157	16014.3 ± 1894.3
mml-miR-148a-5p	243292.7	94 ± 3%	10044.7 ± 3157	16014.3 ± 1894.3
mml-miR-148b-5p	7.70E+02	94 ± 3%	28.4 ± 4	39.2 ± 3.9
mml-miR-150-5p	8.41E+03	94 ± 3%	1019.6 ± 165	644.1 ± 139.6
mml-miR-151-5p	2.10E+04	94 ± 3%	388.3 ± 102.2	556.8 ± 88.6
mml-miR-152-5p	5.5	93 ± 2%	17.3 ± 1.8	25.4 ± 11.1
mml-miR-154-5p	5.2	93 ± 2%	4.6 ± 2	9.6 ± 2.8
mml-miR-155	5.4	93 ± 2%	47.2 ± 24.8	68.3 ± 11.6
mml-miR-15b-5p	1.52E+03	94 ± 3%	95.4 ± 4.1	79.3 ± 7.6
mml-miR-16-5p	180152.8	94 ± 3%	17829.1 ± 2373.4	10801.1 ± 685.6
mml-miR-181a-5p	4.5	85 ± 3%	1745.2 ± 106.2	2061.7 ± 98.1
mml-miR-181b-5p	1.59E+03	94 ± 3%	121.6 ± 18	118.1 ± 7.7
mml-miR-181c-5p	1.69E+03	94 ± 3%	33 ± 14.1	48 ± 13.2
mml-miR-181d	4.09E+02	94 ± 3%	21 ± 6.1	23 ± 5

mml-miR-182	5.4	93 ± 2%	7.7 ± 1.6	3 ± 1
mml-miR-186-5p	5	93 ± 2%	2467.9 ± 322.9	2522.2 ± 437
mml-miR-187-5p	6.31E+03	94 ± 3%	242.1 ± 119.4	241.3 ± 35.8
mml-miR-188-5p	1.09E+02	94 ± 3%	10.6 ± 2.7	7.5 ± 1.6
mml-miR-18b	3.8	58 ± 5%	8.4 ± 1.6	5.4 ± 1
mml-miR-191-5p	210595.2	94 ± 3%	11909.6 ± 2765.7	13502.3 ± 2441.1
mml-miR-192-5p	1.94E+04	94 ± 3%	1981.7 ± 412	1933.7 ± 846.1
mml-miR-194-5p	5.83E+01	94 ± 3%	5.2 ± 1.6	4.1 ± 1.4
mml-miR-195-5p	7.62E+01	94 ± 3%	5.8 ± 1.1	4.8 ± 0.9
mml-miR-196b-5p	4.8	85 ± 3%	2.3 ± 0.6	4 ± 1.1
mml-miR-197-5p	3.51E+03	94 ± 3%	154.9 ± 44.9	240.2 ± 44.7
mml-miR-199a	4.99E+03	94 ± 3%	302.9 ± 43.1	304.4 ± 29.9
mml-miR-199a-5p	4.98E+03	94 ± 3%	302.9 ± 43.1	304.4 ± 29.9
mml-miR-19a-5p	1.03E+03	94 ± 3%	76.2 ± 7.9	64.4 ± 7.5
mml-miR-19b	5.1	93 ± 2%	211.9 ± 28.2	178 ± 15.7
mml-miR-204-5p	1.58E+02	94 ± 3%	12.2 ± 1.6	8.5 ± 2.2
mml-miR-205	5.3	93 ± 2%	17.9 ± 3.4	10.9 ± 3
mml-miR-20a-5p	6.88E+02	94 ± 3%	66 ± 4.2	40.5 ± 4.1
mml-miR-21-5p	4.61E+04	94 ± 3%	1987.4 ± 253.9	3108.8 ± 312.9
mml-miR-214-5p	8.98E+01	94 ± 3%	6.9 ± 1.9	3.1 ± 1
mml-miR-215-5p	3.07E+03	94 ± 3%	424.7 ± 144.5	226.5 ± 82.6
mml-miR-22	473988.3	94 ± 3%	25698.1 ± 2818.2	35319.8 ± 2586.2
mml-miR-221-5p	9.27E+03	94 ± 3%	328.5 ± 28.9	311 ± 35.3
mml-miR-222-5p	3.57E+03	94 ± 3%	225.3 ± 28.1	153 ± 23.9
mml-miR-23a-5p	5.2	93 ± 2%	321.7 ± 27	398.8 ± 29.8
mml-miR-24-3p	5.2	93 ± 2%	42.2 ± 4.9	49.2 ± 6.9
mml-miR-24-5p	4.9	85 ± 3%	42.2 ± 4.9	49.2 ± 6.9
mml-miR-25	3.70E+04	94 ± 3%	4162.1 ± 569.2	2255.2 ± 112.2
mml-miR-26a-5p	2.62E+04	94 ± 3%	1511.8 ± 176.9	2041.6 ± 68.5
mml-miR-26b-5p	2.65E+03	94 ± 3%	134.6 ± 16.7	142.3 ± 11
mml-miR-27a-5p	2.98E+04	94 ± 3%	1311.5 ± 128	1858.1 ± 176.1
mml-miR-27b-5p	7.85E+04	94 ± 3%	3432.7 ± 115	4464.7 ± 420.5

mml-miR-28-5p	1.23E+04	94 ± 3%	603.4 ± 185.6	718.6 ± 109.1
mml-miR-296-5p	0.8	31 ± 4%	7 ± 1.7	3.1 ± 0.9
mml-miR-299-5p	8.68E+02	94 ± 3%	23.9 ± 7.7	45.7 ± 10.6
mml-miR-29a-5p	4.9	85 ± 3%	114.6 ± 21.6	111.5 ± 33.8
mml-miR-29c-5p	5	93 ± 2%	261.8 ± 40	167.3 ± 25.9
mml-miR-301a-5p	4.5	85 ± 3%	36.7 ± 13.8	40.8 ± 8.5
mml-miR-301b	5.4	93 ± 2%	7.8 ± 2.8	8.7 ± 2.7
mml-miR-30a-5p	9.81E+03	94 ± 3%	779.8 ± 136.3	690.2 ± 78.7
mml-miR-30b-5p	1.74E+03	94 ± 3%	93.6 ± 5.4	136.7 ± 13.2
mml-miR-30c-5p	5.32E+03	94 ± 3%	325.6 ± 30.7	422 ± 32.1
mml-miR-30d-5p	1.32E+05	94 ± 3%	6774.6 ± 1624.5	8411.4 ± 1406
mml-miR-30e-5p	7.87E+04	94 ± 3%	4611.4 ± 723.6	4871.1 ± 778
mml-miR-3122	7.61E+01	94 ± 3%	8 ± 2.4	4.1 ± 0.9
mml-miR-32-5p	5.4	93 ± 2%	57.3 ± 10.7	45.7 ± 4.8
mml-miR-320a	5.5	93 ± 2%	493.6 ± 62	372 ± 61.4
mml-miR-320b	4.4	85 ± 3%	315.5 ± 42	250.4 ± 47.4
mml-miR-323a-5p	5.24E+02	94 ± 3%	18.9 ± 6.4	31.1 ± 9.2
mml-miR-324-5p	1.47E+02	94 ± 3%	7.3 ± 1.2	7.1 ± 0.9
mml-miR-330-5p	3.09E+02	94 ± 3%	14.5 ± 4.5	17.5 ± 3.6
mml-miR-331-5p	1.7	57 ± 3%	49.8 ± 5.4	29 ± 1.9
mml-miR-335-5p	2.71E+02	94 ± 3%	26.6 ± 4.2	22 ± 7.7
mml-miR-337-5p	2.34E+02	94 ± 3%	3.4 ± 0.9	7.6 ± 1.3
mml-miR-338-5p	2.71E+02	94 ± 3%	5.7 ± 1.7	4.5 ± 1
mml-miR-339-5p	3.62E+03	94 ± 3%	217.8 ± 30.7	147.4 ± 6.1
mml-miR-33a	5.6	93 ± 2%	2.9 ± 1.1	3.7 ± 0.8
mml-miR-340-5p	3.73E+03	94 ± 3%	145.8 ± 25.1	251.5 ± 41
mml-miR-342-5p	5.4	93 ± 2%	49 ± 8.4	33 ± 8.4
mml-miR-361-5p	2.51E+03	94 ± 3%	110.6 ± 11.6	104.3 ± 16.2
mml-miR-363-5p	4.6	85 ± 3%	29.9 ± 4.6	36.8 ± 6.4
mml-miR-369-5p	2.49E+03	94 ± 3%	57.4 ± 15.7	94.5 ± 24.4
mml-miR-370-3p	0.6	31 ± 4%	24.6 ± 9.5	28.6 ± 8.5
mml-miR-370-5p	5.94E+02	94 ± 3%	24.6 ± 9.5	28.6 ± 8.5

mml-miR-374a-5p	7.53E+01	94 ± 3%	5 ± 0.6	2.7 ± 0.6
mml-miR-374b-5p	7.79E+01	94 ± 3%	3.7 ± 1.4	4.8 ± 1.5
mml-miR-375	1.6	57 ± 3%	345.6 ± 112.7	225.6 ± 99.6
mml-miR-376a-1-5p	1.55E+02	94 ± 3%	3.6 ± 0.8	6.1 ± 1.1
mml-miR-376a-2-5p	1.44E+02	94 ± 3%	3.6 ± 0.8	6.1 ± 1.1
mml-miR-376c-5p	6.48E+02	94 ± 3%	23.5 ± 6	39.8 ± 8.7
mml-miR-377-5p	5.36E+02	94 ± 3%	7 ± 3	12.7 ± 5.5
mml-miR-380-5p	3.9	58 ± 5%	172.7 ± 51.4	364.6 ± 75.7
mml-miR-381-5p	5.3	93 ± 2%	322.1 ± 87.1	389 ± 113.7
mml-miR-382-5p	1.78E+02	94 ± 3%	2.4 ± 1.2	7.2 ± 4.1
mml-miR-409-5p	5.32E+04	94 ± 3%	2029.3 ± 765.3	2885.4 ± 746.3
mml-miR-410-5p	1.45E+04	94 ± 3%	453.4 ± 144.5	944.9 ± 244.3
mml-miR-411-5p	1.90E+03	94 ± 3%	41 ± 4.6	72 ± 12.4
mml-miR-421	3.74E+03	94 ± 3%	144.6 ± 32.1	239.9 ± 36.5
mml-miR-423-5p	236728.2	94 ± 3%	14181.7 ± 1226.2	15446.8 ± 742
mml-miR-429-5p	5.2	93 ± 2%	9 ± 2.4	4.4 ± 1.8
mml-miR-432-5p	1.92E+03	94 ± 3%	59.5 ± 24.2	107.1 ± 41.9
mml-miR-433-5p	1.07E+03	94 ± 3%	41.2 ± 14.7	64 ± 16.6
mml-miR-4446-5p	4.93E+03	94 ± 3%	132.4 ± 51.9	245.7 ± 82.1
mml-miR-453	5.3	93 ± 2%	119.6 ± 40.2	225.5 ± 47.2
mml-miR-4766-5p	5.99E+01	94 ± 3%	3.3 ± 0.7	3.9 ± 1
mml-miR-484	4.9	85 ± 3%	2043.2 ± 52.7	1791.1 ± 160.6
mml-miR-485-5p	2.63E+03	94 ± 3%	80.4 ± 22.2	143.7 ± 37
mml-miR-486-5p	2789851	94 ± 3%	362234.9 ± 75903.4	159672.7 ± 24491.3
mml-miR-487b-5p	2.29E+03	94 ± 3%	38 ± 12.8	72 ± 23.1
mml-miR-493-5p	7.85E+02	94 ± 3%	22.6 ± 7.1	29.7 ± 7.1
mml-miR-494-5p	1.38E+02	94 ± 3%	7.9 ± 2.9	5.7 ± 2.1
mml-miR-495-5p	5	93 ± 2%	4.8 ± 2.6	6.9 ± 2.5
mml-miR-496	1.5	57 ± 3%	2.4 ± 0.9	4.7 ± 0.9
mml-miR-497-5p	2.12E+02	94 ± 3%	19.3 ± 3.2	16.4 ± 3.5
mml-miR-500a-5p	1.71E+02	94 ± 3%	3.4 ± 0.8	17.8 ± 3.9
mml-miR-500b-5p	1.81E+03	94 ± 3%	151.3 ± 15.4	118.1 ± 13.4

mml-miR-501-5p	1.71E+02	94 ± 3%	3.4 ± 0.8	17.8 ± 3.9
mml-miR-502-5p	5.69E+02	94 ± 3%	96.6 ± 9.4	73.8 ± 7.3
mml-miR-505-5p	2.23E+02	94 ± 3%	10.9 ± 1.2	17.8 ± 6.9
mml-miR-532-5p	2.08E+03	94 ± 3%	141.8 ± 14.8	115 ± 13.7
mml-miR-539	3.25E+02	94 ± 3%	8.6 ± 3.1	20.3 ± 4.7
mml-miR-543-5p	2.33E+03	94 ± 3%	54 ± 17.1	104.5 ± 32.1
mml-miR-582-5p	6.47E+01	94 ± 3%	5.2 ± 1.7	1.8 ± 0.8
mml-miR-584-5p	5.7	93 ± 2%	174 ± 42.7	83.9 ± 18.3
mml-miR-590-5p	5.09E+02	94 ± 3%	17.5 ± 5.1	25.7 ± 6.1
mml-miR-598-5p	5.2	93 ± 2%	7.7 ± 2	22.1 ± 8.2
mml-miR-605	5.90E+00	93 ± 2%	61.6 ± 24.8	123.2 ± 24.6
mml-miR-627-5p	4.34E+02	94 ± 3%	27.6 ± 2.6	32.1 ± 2.4
mml-miR-628-5p	3.28E+02	94 ± 3%	15.8 ± 2.8	18.7 ± 2.2
mml-miR-652	5.90E+00	93 ± 2%	50.4 ± 6.1	36.4 ± 4.5
mml-miR-6529-5p	7.14E+04	94 ± 3%	2533.6 ± 793.4	5095.8 ± 694.2
mml-miR-654-5p	5.35E+04	94 ± 3%	1012.6 ± 344.8	1638.8 ± 541.6
mml-miR-656-5p	1.16E+03	94 ± 3%	40.4 ± 9.1	69.1 ± 17.1
mml-miR-660-5p	9.78E+02	94 ± 3%	74.7 ± 10.9	68.5 ± 7.7
mml-miR-665	5.4	93 ± 2%	4.3 ± 1.4	7.5 ± 1
mml-miR-668	5.2	93 ± 2%	6.3 ± 2.3	9.3 ± 3
mml-miR-671-5p	4.54E+02	94 ± 3%	16 ± 6.3	19.6 ± 1.8
mml-miR-6827-5p	1.83E+02	94 ± 3%	6 ± 2.3	12.6 ± 1.2
mml-miR-7	5.4	93 ± 2%	5.1 ± 1.6	7.4 ± 1
mml-miR-7180-5p	3.36E+02	94 ± 3%	15.8 ± 3.9	15.4 ± 2.9
mml-miR-7186-5p	7.39E+02	94 ± 3%	45.4 ± 7.3	31.5 ± 1.4
mml-miR-7205-5p	2.27E+02	94 ± 3%	7.9 ± 2.4	16.8 ± 1
mml-miR-758-5p	1.72E+02	94 ± 3%	3.7 ± 1.1	5.7 ± 1.9
mml-miR-760	4.30E+02	94 ± 3%	18.1 ± 7.1	31.4 ± 2.8
mml-miR-769-5p	4.58E+02	94 ± 3%	21.9 ± 9.1	24.3 ± 6.6
mml-miR-874-5p	1.00E+02	94 ± 3%	11.3 ± 1.9	5.2 ± 1.5
mml-miR-877-5p	1.5	57 ± 3%	31.5 ± 4	30.8 ± 3.8
mml-miR-885-5p	1.59E+02	94 ± 3%	10.2 ± 1.4	20.5 ± 11.9

mml-miR-889-5p	4.78E+03	94 ± 3%	140 ± 46.7	290.3 ± 95.9
mml-miR-92a-3p	650163.6	94 ± 3%	67079.2 ± 8223.8	40718.2 ± 2180.5
mml-miR-92a-5p	626468.8	94 ± 3%	67079.2 ± 8223.8	40718.2 ± 2180.5
mml-miR-93-5p	6.63E+03	94 ± 3%	531.6 ± 49.9	369.3 ± 26.3
mml-miR-942-5p	4.95E+02	94 ± 3%	38.2 ± 3.3	29.9 ± 2.8
mml-miR-98	5.53E+02	94 ± 3%	15.3 ± 3.9	19.6 ± 2.3
mml-miR-99a-5p	8.78E+02	94 ± 3%	106 ± 19	60.8 ± 14.2
mml-miR-99b-5p	6.80E+03	94 ± 3%	648.6 ± 55.6	484.9 ± 42.7

[1]Names of mature miRNAs of Macaca mulatta in miRbase v.20 that match precursor sequences predicted by miRDeep2, and have an randfold p-value < 0.05.
[2]The miRDeep2 score represents the log-odds probability of a sequence being genuine miRNA precursor versus the probability that it is a background hairpin.
[3]The estimated probability that the miRNA candidate is a true positive.
[4]miRNA read counts-per-million computed for each sample and taking into account the estimated dispersions and the libraries sizes. It represents a measure of the average and standard error of the mean expression level of the miRNA in the indicated group.

Table S3 – Abundance (in reads per million) of all circulating novel microRNAs (miRNAs) between CR and control rhesus monkeys.

miRNA unique Id[1]	Precursor coordinate[2]	Human miRBase equivalent[3]	miRDeep2 Score[4]	Estimated probability[5]	Control[6]	CR[6]	FC[7]	P-Value[7]
Novel_chr7_19965	chr7:11722501-11722554	-	0.5	31 ± 4%	128.6 ± 47.8	30.2 ± 4.9	**0.30**	**0.003**
Novel_chr10_2930	chr10:32220145-32220230	-	0.9	31 ± 4%	763 ± 298.8	104 ± 40	**0.19**	**0.004**
Novel_chr11_4054	chr11:20254699-20254744	hsa-miR-4479	1.1	57 ± 3%	746.3 ± 290.9	101.7 ± 39.1	**0.19**	**0.004**
Novel_chr4_17055	chr4:97381342-97381417	-	1.7	57 ± 3%	749.6 ± 292.7	101.9 ± 39	**0.19**	**0.004**
Novel_chr1_989	chr1:194742495-194742554	hsa-miR-3120-3p	269.6	94 ± 3%	6.3 ± 2.1	14.8 ± 1.8	**2.82**	**0.005**
Novel_chr10_3176	chr10:79157581-79157637	hsa-miR-6819-3p	1.6	57 ± 3%	7.8 ± 2.8	18.7 ± 3.4	**2.88**	**0.007**
Novel_chr1_1622	chr1:109272869-109272918	-	2	53 ± 5%	2.1 ± 1.4	7.2 ± 1.2	**3.57**	**0.016**
Novel_chr16_9714	chr16:71213955-71214027	hsa-miR-744-5p	1.7	57 ± 3%	17.5 ± 8	4.4 ± 1	**0.31**	**0.019**
Novel_chr15_8769	chr15:43157172-43157243	-	1.9	57 ± 3%	1.9 ± 0.6	5.3 ± 1.5	**2.94**	**0.03**
Novel_chr14_6523	chr14:10025250-10025311	-	262.8	94 ± 3%	9.5 ± 4.2	20.1 ± 4.1	2.41	0.05
Novel_chr7_19963	chr7:11600504-11600561	hsa-miR-339-5p	0.4	31 ± 4%	41.4 ± 6.7	19 ± 1.8	0.57	0.05
Novel_chr9_23759	chr9:96781413-96781475	hsa-miR-1307-5p	5.2	93 ± 2%	438.8 ± 90.9	687.6 ± 84.4	1.82	0.11
Novel_chr4_16639	chr4:12656370-12656446	hsa-miR-200b-3p	5.2	93 ± 2%	6.3 ± 2.3	2.4 ± 0.6	0.50	0.13
Novel_chr19_11326	chr19:35864834-35864891	hsa-miR-431-5p	3.4	58 ± 5%	60.2 ± 15.3	21.9 ± 10	0.49	0.14
Novel_chr13_5618	chr13:62615584-62615668	-	0.7	31 ± 4%	7.9 ± 2.3	3.1 ± 0.9	0.49	0.15
Novel_chr14_7478	chr14:67823420-67823482	hsa-miR-330-5p	5.7	93 ± 2%	41.2 ± 18.4	65.9 ± 5	1.82	0.16
Novel_chr6_19390	chr6:32640521-32640583	hsa-miR-7155-5p	601.6	94 ± 3%	26.2 ± 6.5	35.5 ± 4.3	1.61	0.17
Novel_chr1_1189	chr1:10872631-10872705	hsa-miR-484	5.5	93 ± 2%	15.6 ± 3.8	8 ± 1	0.64	0.18
Novel_chr1_1190	chr1:10872685-10872732	hsa-miR-484	1	57 ± 3%	15.6 ± 3.8	8 ± 1	0.64	0.19
Novel_chr1_1782	chr1:139164523-139164582	-	0.8	31 ± 4%	77.1 ± 9.8	38.6 ± 7.6	0.63	0.20

Novel_chr20_13518	chr20:11820755-11820816	hsa-miR-5009-5p	0.2	31 ± 4%	379.9 ± 35	482.4 ± 115.7	1.60	0.20
Novel_chr2_13159	chr2:126472529-126472586	-	1.7	57 ± 3%	10.5 ± 3.1	5.1 ± 0.8	0.60	0.21
Novel_chr1_301	chr1:46748582-46748655	hsa-miR-3960	5.9	93 ± 2%	6.8 ± 2.3	3 ± 0.8	0.53	0.21
Novel_chr20_13709	chr20:41814323-41814389	-	1.8	57 ± 3%	3.7 ± 1.4	5.4 ± 1.1	1.77	0.22
Novel_chr7_20224	chr7:67492754-67492823	hsa-miR-3174	8.64E+01	94 ± 3%	3.8 ± 1.3	5.5 ± 1.3	1.74	0.26
Novel_chrX_24467	chrX:58927984-58928048	hsa-miR-1468-5p	4.5	85 ± 3%	2.4 ± 0.9	3.4 ± 0.3	1.66	0.26
Novel_chr3_15025	chr3:183032197-183032258	hsa-miR-3182	1	57 ± 3%	5.2 ± 1.5	7.5 ± 2	1.69	0.27
Novel_chr16_9337	chr16:2072371-2072429	hsa-miR-3182	279.6	94 ± 3%	10.2 ± 2.9	14.6 ± 4.1	1.59	0.28
Novel_chr8_21911	chr8:95882087-95882150	-	1047.5	94 ± 3%	110.9 ± 13.1	62.5 ± 6	0.70	0.28
Novel_chr8_22194	chr8:14596829-14596888	-	0.2	31 ± 4%	11.8 ± 3.3	14.8 ± 3.5	1.48	0.31
Novel_chr20_13868	chr20:74801818-74801879	hsa-miR-3664-3p	1.5	57 ± 3%	11.2 ± 2.9	6.1 ± 0.9	0.69	0.36
Novel_chr7_20515	chr7:140403921-140403966	hsa-miR-6742-5p	2.1	53 ± 5%	20.7 ± 8.9	26.6 ± 3.1	1.47	0.37
Novel_chr8_22307	chr8:39687773-39687846	-	840	94 ± 3%	100.5 ± 31.9	54.5 ± 9.8	0.7	0.37
Novel_chr1_2111	chr1:190862188-190862236	hsa-miR-4802-5p	264.5	94 ± 3%	9 ± 2.6	11.4 ± 2.9	1.42	0.39
Novel_chr9_22986	chr9:55669354-55669418	-	1.04E+02	94 ± 3%	4.3 ± 1.1	5.3 ± 0.6	1.46	0.42
Novel_chr19_11425	chr19:48586236-48586295	-	9.46E+01	94 ± 3%	4.1 ± 0.9	4.7 ± 0.7	1.38	0.44
Novel_chr11_3811	chr11:107963288-107963346	hsa-miR-652-3p	1.41E+02	94 ± 3%	4.7 ± 2.8	7.5 ± 2.8	1.70	0.45
Novel_chr17_10206	chr17:20234014-20234064	hsa-miR-320a	2.9	53 ± 5%	4.5 ± 0.5	5 ± 0.6	1.35	0.47
Novel_chr5_18410	chr5:143938769-143938810	hsa-miR-4286	0	31 ± 4%	16.6 ± 3.3	16.9 ± 2.3	1.22	0.57
Novel_chr4_16920	chr4:54826745-54826806	hsa-miR-4675	5.9	93 ± 2%	4.8 ± 1.5	5.1 ± 0.7	1.22	0.62
Novel_chr5_17463	chr5:38883073-38883131	hsa-miR-574-3p	1020.3	94 ± 3%	77.1 ± 6.5	73.9 ± 5.7	1.17	0.63
Novel_chr5_17997	chr5:16519466-16519544	hsa-miR-1200	2.4	53 ± 5%	5.4 ± 1.3	4.9 ± 2.6	1.13	0.84
Novel_chr19_11313	chr19:33904026-33904084	hsa-miR-4286	1.2	57 ± 3%	4 ± 2.3	3.9 ± 1	1.13	0.93

Novel_chr20_14128	chr20:51463091-51463156	hsa-miR-328-3p	5.9	93 ± 2%	20.3 ± 3.8	16.8 ± 1.9	0.98	0.97
Novel_chr11_4375	chr11:102021020-102021083	hsa-miR-1273h-3p	3	58 ± 5%	4.3 ± 1.3	3.3 ± 0.8	0.99	1.00
Novel_chr19_11778	chr19:41720564-41720615	hsa-miR-320a	4.5	85 ± 3%	10.7 ± 1.3	8.5 ± 1.6	0.98	1.00
Novel_chr20_13867	chr20:74801782-74801841	-	2.2	53 ± 5%	7.3 ± 1.9	6.1 ± 1.1	1.00	1.00

[1]Unique identification containing the chromosome and an arbitrary number assigned to the hairpin predicted by miRDeep2, and have an randfold p-value < 0.05.
[2]Location of the miRNA precursor in the rheMac8 Rhesus genome.
[3]Names of mature miRNAs of the related *Homo sapiens* species in miRbase v.20 (GRCh37.p5) that has the same seed sequence as the candidate mature sequences.
[4]The miRDeep2 score represents the log-odds probability of a sequence being genuine miRNA precursor versus the probability that it is a background hairpin.
[5] The estimated probability that the miRNA candidate is a true positive.
[6]miRNA read counts-per-million computed for each sample and taking into account the estimated dispersions and the libraries sizes. It represents a measure of the average and standard error of the mean expression level of the miRNA in the indicated group.
[7]Fold change and Pvalue for differential abundance were computed by EdgeR from pairwise comparisons for each miRNA between the control and CR groups.

Table S4 – microRNAs differentially expressed between control and CR rhesus monkeys and with significant correlation with body weight, fat percentage or insulin sensitivity.

**microRNAs**	Body weight	Adiposity (%)	Insulin sensitivity
mml-miR-125a-5p	r=0.75, p=0.01	r=0.66, p=0.04	r=-0.64, p=0.05
mml-miR-130a-5p			r=0.77, p=0.01
mml-miR-130b-5p			r=0.81, p=0.01
mml-miR-143-5p	r=0.71, p=0.02		
mml-miR-16-5p	r=0.69, p=0.02		
mml-miR-20a-5p	r=0.66, p=0.04	r=0.68, p=0.03	
mml-miR-337-5p			r=0.80, p=0.01
mml-miR-411-5p			r=0.82, p=0.01
mml-miR-6529-5p	r=-0.68, p=0.05		r=0.67, p=0.05
mml-miR-92a-3p			r=-0.71, p=0.03

* r values and p values calculated by linear regressions

Table S5 – Known and novel microRNAs non-responsive to CR, but with significant correlation with body weight, fat percentage or insulin.

microRNAs	Body weight (kg)	Fat (%)	Insulin sensitivity
**Known miRNAs**	r value, p value*	r value, p value*	r value, p value*
miR-10a-5p	r=0.69, p=0.02		
miR-10b-5p	r=0.72, p=0.02		
miR-1260b-5p	r=-0.71, p=0.02	r=0.41, p=0.06	
miR-136			r=0.71, p=0.02
miR-181a-5p	r=-0.68, p=0.03	r=-0.66, p=0.04	
miR-182-5p	r=0.66, p=0.04		
miR-197-5p	r=-0.69, p=0.03	r=-0.64, p=0.05	
miR-205-5p	r=0.74, p=0.01		
miR-214-5p	r=0.64, p=0.05		
miR-22			r=0.73, p=0.02
miR-26a-5p	r=-0.93, p<0.01	r=-0.90, p<0.01	
miR-26b-5p	r=-0.72, p=0.02		
miR-29c-5p	r=0.67, p=0.03		
miR-30b-5p	r=-0.74, p=0.01	r=-0.76, p<0.01	
miR-30b-5p	r=-0.74, p=0.01	r=-0.78, p<0.01	
miR-32-5p	r=0.79, p<0.01		
miR-376a1-5p	r=-0.73, p=0.01	r=-0.70, p=0.02	r=0.75, p=0.02
miR-376a2-5p	r=-0.73, p=0.01	r=-0.70, p=0.02	r=0.75, p=0.02
miR-380-5p			r=0.71, p=0.03
miR-539			r=0.71, p=0.03
miR-584-5p	r=0.73, p=0.02		
miR-590-5p	r=-0.67, p=0.03		
miR-671-5p	r=-0.63, p=0.04		
miR-6827-5p	r=-0.72, p=0.02	r=-0.69, p=0.03	
miR-7-5p	r=-0.77, p<0.01	r=-0.68, p=0.03	
miR-7186-5p	r=0.65, p=0.04		
miR-7205-5p	r=-0.80, p=0.004	r=-0.78, p=0.007	r=0.71, p=0.03
miR-760-5p	r=-0.68, p=0.03		
miR-92a-5p	r=-0.71, p=0.03		
miR-99b-5p	r=0.65, p=0.04		
**Novel miRNAs**			
Novel_chr1_989			r=0.73, p=0.02
Novel_chr10_3176	r=-0.92, p<0.01	r=-0.92, p<0.01	r=0.66, p=0.05
Novel_chr1_1622	r=-0.62, p=0.03		

* r values and p values calculated by linear regressions

Table S6 - Enriched KEGG pathways for the genes targeted by miRNA differentially expressed between CR and control rhesus monkeys.

Term	Count	%	p Value	Fold Enrichment	Benjamini
hsa03010:Ribosome	59	2.01	2.35E-11	2.37	6.87E-09
**hsa04110:Cell cycle**	**52**	**1.77**	**3.35E-09**	**2.26**	**4.90E-07**
hsa05205:Proteoglycans in cancer	69	2.35	6.21E-08	1.88	6.04E-06
hsa05215:Prostate cancer	38	1.29	1.22E-07	2.38	8.90E-06
hsa03013:RNA transport	61	2.08	1.59E-07	1.92	9.31E-06
hsa05220:Chronic myeloid leukemia	33	1.12	3.54E-07	2.47	1.72E-05
hsa05200:Pathways in cancer	112	3.82	8.91E-07	1.53	3.72E-05
hsa05169:Epstein-Barr virus infection	63	2.15	1.46E-06	1.79	5.32E-05
hsa05212:Pancreatic cancer	29	0.99	2.80E-06	2.44	9.07E-05
**hsa04115:p53 signaling pathway**	**29**	**0.99**	**5.81E-06**	**2.37**	**1.54E-04**
**hsa04068:FoxO signaling pathway**	**47**	**1.60**	**5.50E-06**	**1.92**	**1.61E-04**
hsa04066:HIF-1 signaling pathway	37	1.26	1.53E-05	2.03	3.71E-04
hsa05230:Central carbon metabolism in cancer	27	0.92	3.13E-05	2.27	7.04E-04
hsa05223:Non-small cell lung cancer	24	0.82	7.26E-05	2.31	0.001
hsa05161:Hepatitis B	47	1.60	6.88E-05	1.76	0.001
hsa04919:Thyroid hormone signaling pathway	39	1.33	1.06E-04	1.84	0.002
hsa05214:Glioma	25	0.85	2.07E-04	2.14	0.003
hsa05203:Viral carcinogenesis	60	2.04	1.96E-04	1.58	0.003
hsa04012:ErbB signaling pathway	31	1.06	2.85E-04	1.92	0.004
hsa03040:Spliceosome	42	1.43	2.75E-04	1.73	0.004
hsa04550:Signaling pathways regulating pluripotency of stem cells	44	1.50	3.12E-04	1.69	0.004
hsa00020:Citrate cycle (TCA cycle)	15	0.51	3.97E-04	2.69	0.005
hsa05222:Small cell lung cancer	30	1.02	4.38E-04	1.90	0.005
hsa01200:Carbon metabolism	37	1.26	3.60E-04	1.78	0.005
hsa04141:Protein processing in endoplasmic reticulum	50	1.70	4.10E-04	1.61	0.005
**hsa04151:PI3K-Akt signaling pathway**	**90**	**3.07**	**4.03E-04**	**1.40**	**0.005**
hsa00310:Lysine degradation	21	0.72	5.95E-04	2.17	0.006
**hsa04931:Insulin resistance**	**35**	**1.19**	**7.90E-04**	**1.74**	**0.008**
hsa05211:Renal cell carcinoma	24	0.82	9.44E-04	1.99	0.009
hsa05202:Transcriptional misregulation in cancer	49	1.67	8.97E-04	1.57	0.009

Table S7 - Enriched gene ontology (GO) terms for molecular function and biological process for the genes targeted by miRNA differentially expressed between calorie restricted and control rhesus monkeys.

Term	Count	%	P Value	Fold Enrichment	Benjamini
**GO Terms Molecular function**					
GO:0044822~poly(A) RNA binding	430	14.65	3.88E-76	2.35	6.94E-73
GO:0005515~protein binding	1819	61.98	1.56E-64	1.28	1.39E-61
GO:0005524~ATP binding	364	12.40	1.22E-18	1.52	7.27E-16
GO:0003723~RNA binding	133	4.53	6.73E-13	1.83	3.02E-10
GO:0031625~ubiquitin protein ligase binding	89	3.03	2.04E-12	2.10	7.29E-10
GO:0003735~structural constituent of ribosome	74	2.52	4.60E-11	2.16	1.37E-08
GO:0003682~chromatin binding	112	3.82	6.36E-11	1.83	1.63E-08
GO:0019899~enzyme binding	99	3.37	3.32E-10	1.86	7.44E-08
GO:0008134~transcription factor binding	89	3.03	4.94E-10	1.92	9.83E-08
GO:0019901~protein kinase binding	109	3.71	2.46E-09	1.74	4.41E-07
GO:0051082~unfolded protein binding	39	1.33	1.96E-07	2.36	3.19E-05
GO:0042826~histone deacetylase binding	39	1.33	4.69E-07	2.30	7.00E-05
GO:0031072~heat shock protein binding	23	0.78	6.35E-07	3.06	8.75E-05
GO:0016887~ATPase activity	51	1.74	8.65E-07	2.00	1.11E-04
GO:0008017~microtubule binding	57	1.94	4.61E-06	1.83	5.50E-04
GO:0003743~translation initiation factor activity	23	0.78	5.45E-06	2.76	6.10E-04
GO:0000166~nucleotide binding	82	2.79	1.08E-05	1.60	0.001
GO:0004672~protein kinase activity	57	1.94	1.08E-05	1.78	0.001
GO:0004004~ATP-dependent RNA helicase activity	26	0.89	1.08E-05	2.49	0.001
GO:0002039~p53 binding	27	0.92	1.15E-05	2.43	0.001
GO:0003730~mRNA 3'-UTR binding	20	0.68	1.56E-05	2.85	0.001
GO:0043022~ribosome binding	19	0.65	1.91E-05	2.91	0.002
GO:0003713~transcription coactivator activity	66	2.25	2.66E-05	1.66	0.002
GO:0008022~protein C-terminus binding	53	1.81	6.61E-05	1.72	0.005
GO:0032947~protein complex scaffold	18	0.61	1.15E-04	2.69	0.008
GO:0042802~identical protein binding	132	4.50	1.30E-04	1.36	0.009
**GO Terms Biological Process**					
GO:0010467~gene expression	329	11.21	7.59E-45	2.10	4.59E-41
GO:0016032~viral process	191	6.51	3.05E-27	2.15	9.24E-24

GO:0006413~translational initiation	77	2.62	1.22E-26	3.52	2.47E-23
GO:0006412~translation	113	3.85	4.87E-21	2.43	7.37E-18
GO:0019083~viral transcription	61	2.08	6.52E-20	3.36	7.89E-17
GO:0000184~nuclear-transcribed mRNA catabolic process, nonsense-mediated decay	63	2.15	1.08E-19	3.27	1.08E-16
GO:0019058~viral life cycle	73	2.49	1.65E-19	2.96	1.43E-16
GO:0006414~translational elongation	55	1.87	7.69E-19	3.47	5.82E-16
GO:0006614~SRP-dependent cotranslational protein targeting to membrane	57	1.94	7.90E-18	3.26	5.31E-15
GO:0000278~mitotic cell cycle	140	4.77	3.80E-17	2.00	2.30E-14
GO:0016259~selenocysteine metabolic process	49	1.67	1.97E-16	3.41	1.22E-13
GO:0006415~translational termination	48	1.64	1.97E-16	3.45	1.12E-13
GO:0044267~cellular protein metabolic process	238	8.11	2.56E-16	1.65	1.03E-13
GO:0001887~selenium compound metabolic process	56	1.91	1.19E-15	3.03	5.28E-13
GO:0044281~small molecule metabolic process	385	13.12	1.44E-12	1.38	5.82E-10
GO:0034641~cellular nitrogen compound metabolic process	103	3.51	7.53E-12	1.94	2.85E-09
GO:0045944~positive regulation of transcription from RNA polymerase II promoter	238	8.11	1.02E-11	1.51	3.64E-09
GO:0008380~RNA splicing	84	2.86	1.34E-11	2.09	4.51E-09
GO:0051301~cell division	97	3.30	1.45E-09	1.83	4.61E-07
GO:0007050~cell cycle arrest	53	1.81	4.82E-09	2.27	1.46E-06
GO:0050821~protein stabilization	47	1.60	4.20E-08	2.26	1.21E-05
GO:0000398~mRNA splicing, via spliceosome	66	2.25	5.19E-08	1.95	1.43E-05
GO:0043066~negative regulation of apoptotic process	125	4.26	1.39E-07	1.57	3.67E-05
GO:0045893~positive regulation of transcription, DNA-templated	127	4.33	1.42E-07	1.56	3.58E-05
GO:0006325~chromatin organization	72	2.45	1.52E-07	1.84	3.67E-05
GO:0006367~transcription initiation from RNA polymerase II promoter	74	2.52	2.57E-07	1.80	5.99E-05
GO:0016925~protein sumoylation	42	1.43	2.80E-07	2.25	6.28E-05
GO:0097193~intrinsic apoptotic signaling pathway	27	0.92	4.75E-07	2.80	1.03E-04
GO:0000122~negative regulation of transcription from RNA polymerase II promoter	169	5.76	4.91E-07	1.43	1.02E-04
GO:0006974~cellular response to DNA damage stimulus	61	2.08	5.39E-07	1.89	1.09E-04
GO:1900034~regulation of cellular response to heat	31	1.06	8.25E-07	2.53	1.61E-04
GO:0007264~small GTPase mediated signal transduction	165	5.62	8.98E-07	1.43	1.70E-04
GO:0007179~transforming growth factor beta receptor signaling pathway	46	1.57	9.07E-07	2.08	1.66E-04
GO:0000082~G1/S transition of mitotic cell cycle	50	1.70	9.32E-07	2.01	1.66E-04
GO:0007077~mitotic nuclear envelope disassembly	22	0.75	1.20E-06	3.06	2.08E-04
GO:0006915~apoptotic process	143	4.87	1.27E-06	1.46	2.13E-04

GO:0051028~mRNA transport	26	0.89	1.91E-06	2.70	3.13E-04
GO:0012501~programmed cell death	50	1.70	3.25E-06	1.94	5.17E-04
GO:0046777~protein autophosphorylation	52	1.77	3.41E-06	1.90	5.29E-04
GO:0071407~cellular response to organic cyclic compound	25	0.85	3.50E-06	2.68	5.30E-04
GO:0034605~cellular response to heat	38	1.29	3.98E-06	2.15	5.87E-04
GO:0045429~positive regulation of nitric oxide biosynthetic process	21	0.72	5.54E-06	2.92	7.98E-04
GO:0006396~RNA processing	29	0.99	6.84E-06	2.40	9.63E-04
GO:0043488~regulation of mRNA stability	35	1.19	9.09E-06	2.16	0.001
GO:0006418~tRNA aminoacylation for protein translation	20	0.68	1.03E-05	2.91	0.001
GO:0001934~positive regulation of protein phosphorylation	42	1.43	1.27E-05	1.98	0.002
GO:0006468~protein phosphorylation	108	3.68	1.76E-05	1.48	0.002
GO:0071456~cellular response to hypoxia	39	1.33	1.90E-05	2.00	0.002
GO:0008285~negative regulation of cell proliferation	98	3.34	2.00E-05	1.51	0.002
GO:0006461~protein complex assembly	38	1.29	2.02E-05	2.02	0.002
GO:0048013~ephrin receptor signaling pathway	33	1.12	2.02E-05	2.15	0.002
GO:0002181~cytoplasmic translation	12	0.41	2.25E-05	4.08	0.003
GO:0000462~maturation of SSU-rRNA from tricistronic rRNA transcript (SSU-rRNA, 5.8S rRNA, LSU-rRNA)	13	0.44	2.43E-05	3.79	0.003
GO:0048011~neurotrophin TRK receptor signaling pathway	97	3.30	2.48E-05	1.50	0.003
GO:0006281~DNA repair	94	3.20	2.49E-05	1.51	0.003
GO:0001889~liver development	33	1.12	3.26E-05	2.10	0.004
GO:0007173~epidermal growth factor receptor signaling pathway	82	2.79	3.29E-05	1.55	0.003
GO:0034644~cellular response to UV	20	0.68	3.43E-05	2.72	0.004
GO:0006397~mRNA processing	48	1.64	3.54E-05	1.81	0.004
GO:0006457~protein folding	51	1.74	3.73E-05	1.77	0.004
GO:1900182~positive regulation of protein localization to nucleus	11	0.37	4.11E-05	4.21	0.004
GO:0045892~negative regulation of transcription, DNA-templated	113	3.85	4.88E-05	1.43	0.005
GO:0001525~angiogenesis	60	2.04	5.06E-05	1.67	0.005
GO:0001843~neural tube closure	28	0.95	6.29E-05	2.20	0.006
GO:0007219~Notch signaling pathway	47	1.60	6.44E-05	1.79	0.006
GO:0006446~regulation of translational initiation	17	0.58	6.72E-05	2.89	0.006
GO:0006366~transcription from RNA polymerase II promoter	126	4.29	6.84E-05	1.39	0.006
GO:0033138~positive regulation of peptidyl-serine phosphorylation	26	0.89	8.46E-05	2.24	0.007
GO:0035264~multicellular organism growth	30	1.02	9.47E-05	2.09	0.008
GO:0016568~chromatin modification	32	1.09	1.06E-04	2.02	0.009

GO:0051726~regulation of cell cycle	37	1.26	1.10E-04	1.90	0.009

Table S8 – Functional Annotation Cluster reporting terms for which contributing terms were independently significant for the genes targeted by miRNA differentially expressed between CR and control rhesus monkeys.

Category	Term	Count	%	P Value	Fold Enric.	Benjamini
**Annotation Cluster 1**	**Enrichment Score: 16.23**					
GOTERM_BP_DIRECT	GO:0006413~translational initiation	77	2.62	1.22E-26	3.52	2.47E-23
GOTERM_BP_DIRECT	GO:0006412~translation	113	3.85	4.87E-21	2.43	7.37E-18
UP_KEYWORDS	Ribonucleoprotein	105	3.58	3.19E-20	2.51	1.62E-18
GOTERM_BP_DIRECT	GO:0019083~viral transcription	61	2.08	6.52E-20	3.36	7.89E-17
GOTERM_BP_DIRECT	GO:0000184~nuclear-transcribed mRNA catabolic process, nonsense-mediated decay	63	2.15	1.08E-19	3.27	1.08E-16
GOTERM_BP_DIRECT	GO:0019058~viral life cycle	73	2.49	1.65E-19	2.96	1.43E-16
GOTERM_BP_DIRECT	GO:0006414~translational elongation	55	1.87	7.69E-19	3.47	5.82E-16
GOTERM_BP_DIRECT	GO:0006614~SRP-dependent cotranslational protein targeting to membrane	57	1.94	7.90E-18	3.26	5.31E-15
GOTERM_BP_DIRECT	GO:0016259~selenocysteine metabolic process	49	1.67	1.97E-16	3.41	1.22E-13
GOTERM_BP_DIRECT	GO:0006415~translational termination	48	1.64	1.97E-16	3.45	1.12E-13
GOTERM_BP_DIRECT	GO:0001887~selenium compound metabolic process	56	1.91	1.19E-15	3.03	5.28E-13
UP_KEYWORDS	Ribosomal protein	67	2.28	3.86E-13	2.51	1.27E-11
GOTERM_BP_DIRECT	GO:0034641~cellular nitrogen compound metabolic process	103	3.51	7.53E-12	1.94	2.85E-09
GOTERM_CC_DIRECT	GO:0022625~cytosolic large ribosomal subunit	33	1.12	9.82E-12	3.56	8.66E-10
KEGG_PATHWAY	hsa03010:Ribosome	59	2.01	2.35E-11	2.37	6.87E-09
GOTERM_MF_DIRECT	GO:0003735~structural constituent of ribosome	74	2.52	4.60E-11	2.16	1.37E-08
**Annotation Cluster 2**	**Enrichment Score: 10.67**					
UP_KEYWORDS	Cell cycle	181	6.17	1.13E-19	1.94	5.25E-18
UP_KEYWORDS	Cell division	110	3.75	5.06E-13	1.99	1.57E-11
UP_KEYWORDS	Mitosis	77	2.62	3.87E-10	2.06	7.71E-09
GOTERM_BP_DIRECT	GO:0051301~cell division	97	3.30	1.45E-09	1.83	4.61E-07
GOTERM_BP_DIRECT	GO:0007067~mitotic nuclear division	63	2.15	1.39E-04	1.59	0.011436
**Annotation Cluster 3**	**Enrichment Score: 8.91**					
UP_KEYWORDS	Nucleotide-binding	427	14.55	8.85E-29	1.65	6.18E-27
UP_KEYWORDS	ATP-binding	349	11.89	3.19E-27	1.73	1.98E-25
GOTERM_MF_DIRECT	GO:0005524~ATP binding	364	12.40	1.22E-18	1.52	7.27E-16
UP_SEQ_FEATURE	nucleotide phosphate-binding region:ATP	246	8.38	2.25E-18	1.70	7.32E-15

UP_SEQ_FEATURE	binding site:ATP	135	4.60	1.30E-09	1.66	2.12E-06
UP_KEYWORDS	Kinase	163	5.55	1.62E-09	1.57	2.83E-08
UP_SEQ_FEATURE	domain:Protein kinase	112	3.82	2.17E-07	1.61	1.18E-04
UP_SEQ_FEATURE	active site:Proton acceptor	146	4.97	3.94E-07	1.49	1.71E-04
INTERPRO	IPR011009:Protein kinase-like domain	124	4.22	1.42E-06	1.51	5.18E-04
INTERPRO	IPR000719:Protein kinase, catalytic domain	115	3.92	1.99E-06	1.53	6.60E-04
GOTERM_BP_DIRECT	GO:0046777~protein autophosphorylation	52	1.77	3.41E-06	1.90	5.29E-04
UP_KEYWORDS	Serine/threonine-protein kinase	92	3.13	4.25E-06	1.60	4.65E-05
GOTERM_MF_DIRECT	GO:0004672~protein kinase activity	57	1.94	1.08E-05	1.78	0.001
GOTERM_BP_DIRECT	GO:0006468~protein phosphorylation	108	3.68	1.76E-05	1.48	0.002
INTERPRO	IPR017441:Protein kinase, ATP binding site	90	3.07	3.05E-05	1.53	0.007
INTERPRO	IPR008271:Serine/threonine-protein kinase, active site	73	2.49	2.46E-04	1.51	0.044
GOTERM_MF_DIRECT	GO:0004674~protein serine/threonine kinase activity	79	2.69	3.79E-04	1.46	0.020
INTERPRO	IPR002290:Serine/threonine- / dual specificity protein kinase, catalytic domain	62	2.11	1.78E-03	1.46	0.184
SMART	SM00220:SM00220	62	2.11	7.87E-03	1.37	0.329
**Annotation Cluster 4**	**Enrichment Score: 8.43**					
UP_KEYWORDS	Mitochondrion	246	8.38	2.04E-12	1.53	5.70E-11
UP_KEYWORDS	Transit peptide	130	4.43	1.95E-09	1.67	3.29E-08
UP_SEQ_FEATURE	transit peptide:Mitochondrion	118	4.02	7.64E-09	1.68	9.95E-06
GOTERM_CC_DIRECT	GO:0005759~mitochondrial matrix	81	2.76	6.20E-06	1.63	2.73E-04
**Annotation Cluster 5**	**Enrichment Score: 8.40**					
UP_KEYWORDS	mRNA splicing	86	2.93	6.68E-14	2.28	2.49E-12
UP_KEYWORDS	mRNA processing	100	3.41	1.92E-13	2.10	6.69E-12
GOTERM_BP_DIRECT	GO:0008380~RNA splicing	84	2.86	1.34E-11	2.09	4.51E-09
UP_KEYWORDS	Spliceosome	46	1.57	3.88E-09	2.48	6.02E-08
GOTERM_BP_DIRECT	GO:0000398~mRNA splicing, via spliceosome	66	2.25	5.19E-08	1.95	1.43E-05
GOTERM_CC_DIRECT	GO:0071013~catalytic step 2 spliceosome	35	1.19	1.63E-07	2.53	1.05E-05
GOTERM_BP_DIRECT	GO:0006397~mRNA processing	48	1.64	3.54E-05	1.81	0.004
KEGG_PATHWAY	hsa03040:Spliceosome	42	1.43	2.75E-04	1.73	0.004

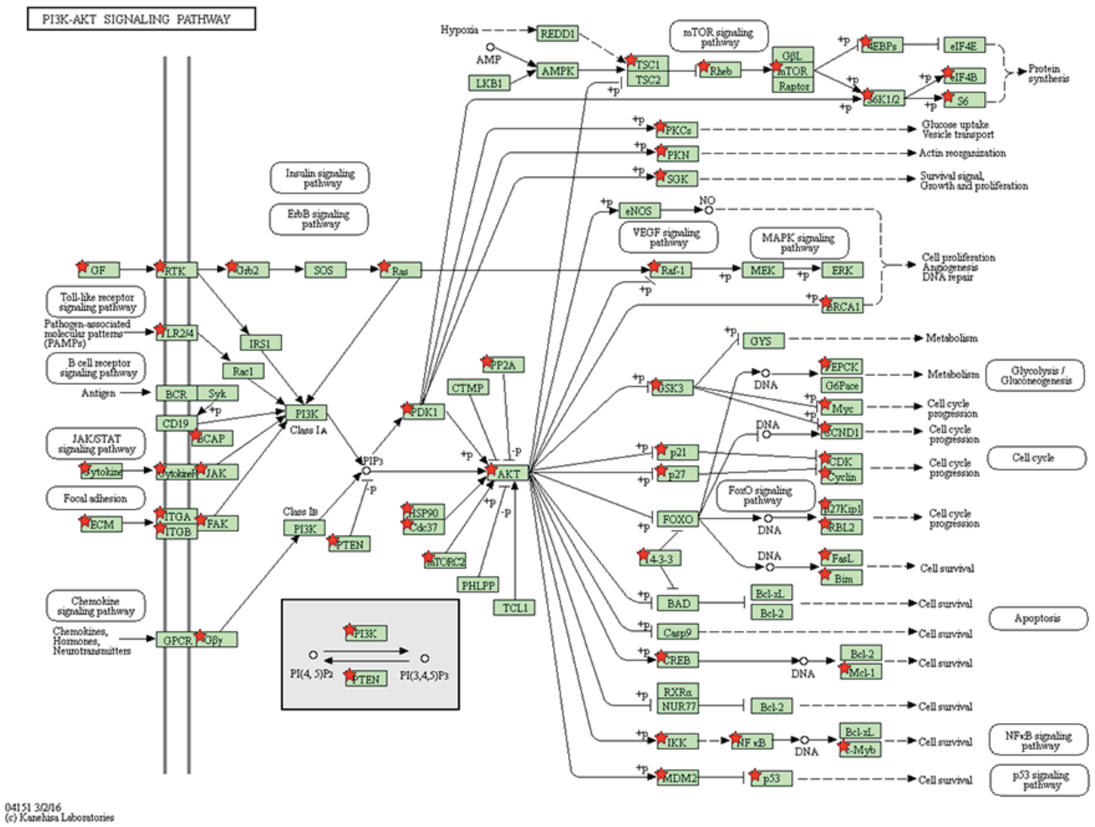

Figure S1. PI3K-AKT signaling pathway. Red stars identify gene targets of CR-regulated miRNAs. This pathway was identified using DAVID to probe the KEGG database. Image used with permission from KEGG Database.
